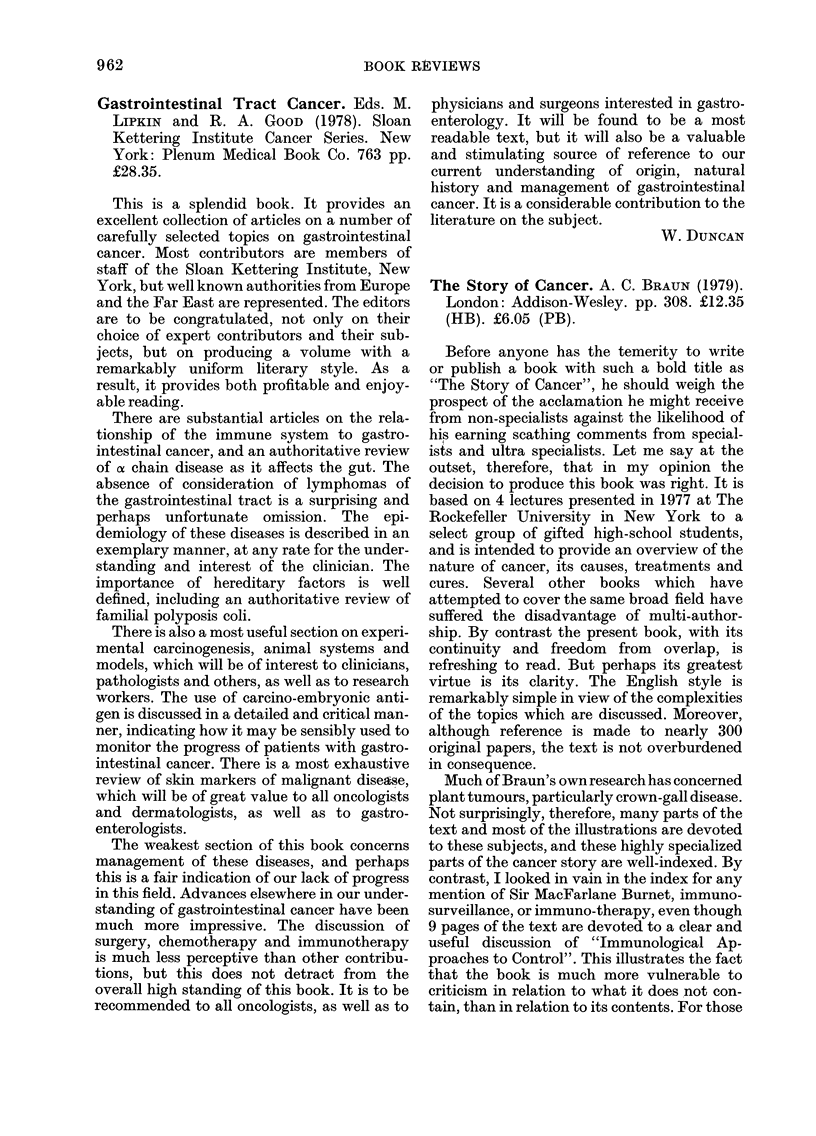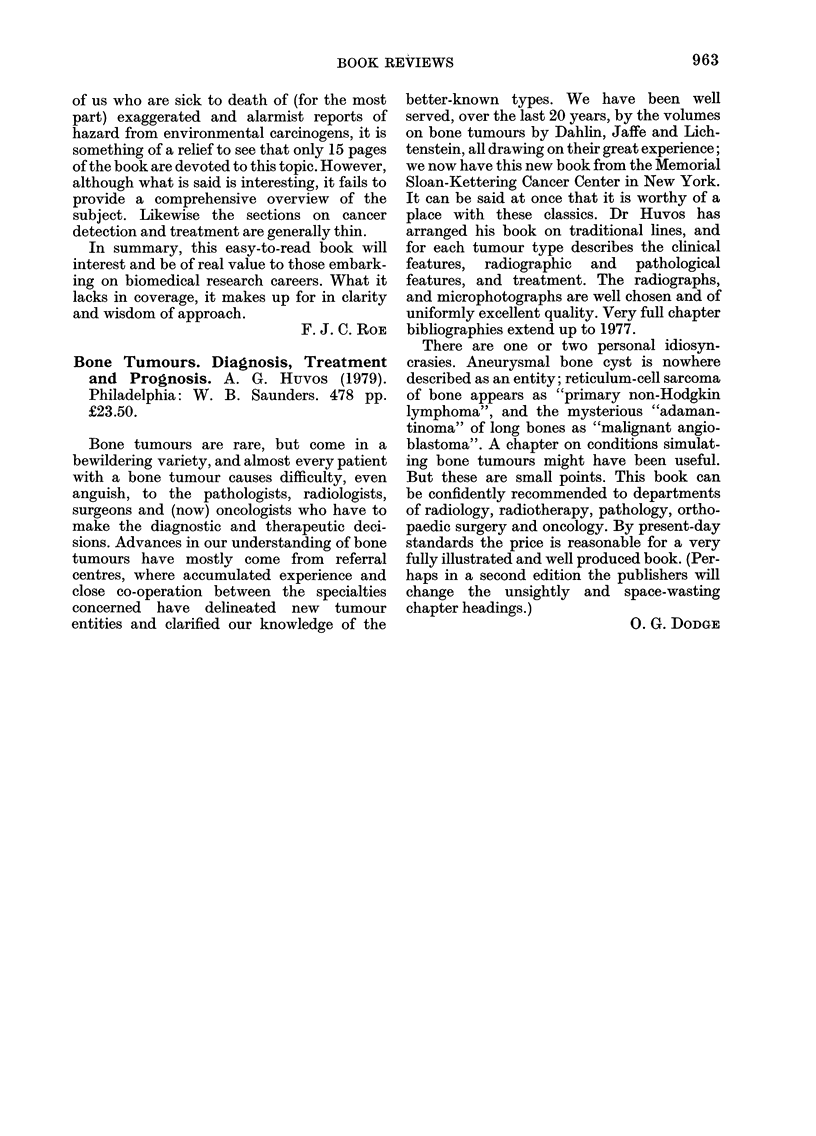# The Story of Cancer

**Published:** 1979-12

**Authors:** F. J. C. Roe


**The Story of Cancer.** A. C. Braun (1979). London: Addison-Wesley. pp. 308. £12.35 (HB). £6.05 (PB).

Before anyone has the temerity to write or publish a book with such a bold title as "The Story of Cancer", he should weigh the prospect of the acclamation he might receive from non-specialists against the likelihood of his earning scathing comments from specialists and ultra specialists. Let me say at the outset, therefore, that in my opinion the decision to produce this book was right. It is based on 4 lectures presented in 1977 at The Rockefeller University in New York to a select group of gifted high-school students, and is intended to provide an overview of the nature of cancer, its causes, treatments and cures. Several other books which have attempted to cover the same broad field have suffered the disadvantage of multi-authorship. By contrast the present book, with its continuity and freedom from overlap, is refreshing to read. But perhaps its greatest virtue is its clarity. The English style is remarkably simple in view of the complexities of the topics which are discussed. Moreover, although reference is made to nearly 300 original papers, the text is not overburdened in consequence.

Much of Braun's own research has concerned plant tumours, particularly crown-gall disease. Not surprisingly, therefore, many parts of the text and most of the illustrations are devoted to these subjects, and these highly specialized parts of the cancer story are well-indexed. By contrast, I looked in vain in the index for any mention of Sir MacFarlane Burnet, immunosurveillance, or immuno-therapy, even though 9 pages of the text are devoted to a clear and useful discussion of "Immunological Approaches to Control". This illustrates the fact that the book is much more vulnerable to criticism in relation to what it does not contain, than in relation to its contents. For those of us who are sick to death of (for the most part) exaggerated and alarmist reports of hazard from environmental carcinogens, it is something of a relief to see that only 15 pages of the book are devoted to this topic. However, although what is said is interesting, it fails to provide a comprehensive overview of the subject. Likewise the sections on cancer detection and treatment are generally thin.

In summary, this easy-to-read book will interest and be of real value to those embarking on biomedical research careers. What it lacks in coverage, it makes up for in clarity and wisdom of approach.

F. J. C. Roe